# A critical review of systematic reviews and meta-analyses of curcumin for knee osteoarthritis

**DOI:** 10.3389/fphar.2025.1664319

**Published:** 2026-01-05

**Authors:** Jixin Chen, Qinxin Zhou, Weijie Yu, Dongdong Cao, Yingzhou Li, Jianliang Chen, Feng Ye

**Affiliations:** 1 Department of Orthopaedic Surgery, Shaoxing Shangyu District Hospital of Traditional Chinese Medicine, Shaoxing, China; 2 Department of Orthopaedic Surgery, Shaoxing Hospital of Traditional Chinese Medicine, shaoxing, China; 3 Department of Orthopaedic Surgery, Yunnan Provincial Hospital of Chinese Medicine, Kunming, China; 4 Department of Orthopaedic Surgery, First Teaching Hospital of Tianjin University of Traditional Chinese Medicine, Tianjin, China

**Keywords:** curcumin, knee osteoarthritis, overview, systematic review, methodological quality, meta-analysis

## Abstract

**Objective:**

This study aims to assess the efficacy of curcumins in treating knee osteoarthritis (KOA).

**Methods:**

We performed a comprehensive review of literature from inception to 11 January 2025, identifying all systematic reviews (SRs) and meta-analyses (MAs) on curcumin treatment for KOA. Two independent reviewers conducted literature screening and data extraction. Evaluation of methodological and reporting quality, risk of bias, evidence quality, and evidence overlap was carried out using AMSTAR 2, PRISMA 2020, ROBIS, GRADE, and GROOVE frameworks.

**Results:**

Seven SRs met inclusion criteria. Curcumins demonstrated potential efficacy and safety advantages over control treatments in KOA management. However, these reviews were of extremely low methodological quality, with poor reporting and significant information gaps. High risk of bias was noted in four SRs. Among 48 outcomes assessed, evidence quality was mostly low, with 5 medium-quality, 6 low-quality, and 37 extremely low-quality evidences. Significant literature overlap was evident.

**Conclusion:**

The current SRs on curcumins for KOA are of low quality. Future research should adhere to rigorous quality assessment standards, increase sample sizes to minimize overlap, and thoroughly evaluate evidence quality to enhance the reliability and rigor of evidence supporting clinical practice.

**Systematic Review Registration:**

https://www.crd.york.ac.uk/prospero/display_record.php?ID=CRD42025641801, identifier CRD42025641801.

## 1 Introduction

Knee osteoarthritis (KOA) is a chronic degenerative joint disorder that affects millions of individuals worldwide, particularly older adults. It is characterized by progressive cartilage degradation, subchondral bone changes, and synovial inflammation, leading to pain, stiffness, and functional impairment (Laslett et al., 2024). The condition not only diminishes quality of life but also imposes a significant economic and healthcare burden. Current treatments for KOA focus primarily on managing symptoms rather than reversing disease progression. Pharmacological options, such as non-steroidal anti-inflammatory drugs (NSAIDs), intra-articular corticosteroids, and hyaluronic acid injections, offer temporary relief but are associated with significant risks, including gastrointestinal, cardiovascular, and renal complications with long-term use (Katz et al., 2021). Surgical interventions, such as knee replacement, are reserved for advanced cases but carry high costs and the risk of complications. These limitations highlight the need for safer, more effective therapeutic alternatives (Feki et al., 2024).

Curcumin, a bioactive compound derived from the rhizome of Curcuma longa (turmeric), has gained attention for its anti-inflammatory and antioxidant properties (Zhao et al., 2024). It acts by inhibiting the nuclear factor kappa-B (NF-κB) signaling pathway and reducing the production of inflammatory cytokines, such as tumor necrosis factor-α (TNF-α) and interleukin-1β (IL-1β). Additionally, curcumin mitigates oxidative stress by scavenging reactive oxygen species (ROS), thereby protecting cartilage from degradation (Chin, 2016). Clinical studies suggest that curcumin can significantly improve pain scores and joint functionality in KOA patients. It has shown comparable efficacy to NSAIDs in pain relief but with fewer adverse effects, making it a potentially safer alternative (Mahdi et al., 2020). However, questions remain regarding the optimal dosage, treatment duration, and long-term efficacy of curcumin.

Despite its potential, the efficacy of curcumin in treating KOA remains a subject of debate. While some systematic reviews (SRs) and meta-analyses (MAs) report significant benefits, others highlight inconsistencies in study quality, small sample sizes, and heterogeneity in outcomes. These issues make it difficult to draw definitive conclusions about its therapeutic value. To address these challenges, this study conducts an umbrella review of existing systematic reviews and meta-analyses using established methodologies, including A MeaSurement Tool to Assess systematic Reviews 2 (AMSTAR 2), Preferred Reporting Items for Systematic Reviews and Meta-Analyses 2020 (PRISMA 2020), Risk Of Bias In Systematic reviews (ROBIS), Grading of Recommendations Assessment, Development and Evaluation (GRADE), and Graphical Representation of Overlap for OVErviews (GROOVE). By synthesizing qualitative evidence, this research aims to provide a comprehensive evaluation of curcumin’s role in managing KOA symptoms and to identify directions for future studies.

## 2 Methods and materials

### 2.1 Protocol and registration

This study provides a comprehensive analysis of SRs and MAs on the use of curcumin in the treatment of KOA. The methodological quality and evidence reliability were assessed using the “Preferred Reporting Items for Overviews of Reviews” (PRIOR) framework (Pollock et al., 2019), the PRISMA guidelines, and the AMSTAR 2 tool. Detailed methodology can be found in the Supplementary Materials. Furthermore, this study has been registered on the PROSPERO platform under registration number CRD42025641801.

### 2.2 Search strategy

A comprehensive literature search was independently conducted by two reviewers across eight databases, including four English-language databases (Cochrane Library, EMBASE, PubMed, and Web of Science) and four Chinese-language databases (CNKI, CBM, WanFang, and VIP). The search covered publications up to 11 January 2025. The exact date of the last search performed was 11 January 2025. The search was supplemented by manual reviews of grey literature and consultations with field experts. No restrictions were applied regarding language or publication location. The search strategy combined subject-specific terms with free-text keywords, such as “knee osteoarthritis,” “curcumin,” “curcuma,” “meta-analysis,” and “systematic review.” Full details of the database-specific search processes can be found in the Supplementary Materials.

### 2.3 Inclusion and exclusion criteria

The study selection focused on SRs or MAs addressing curcumin treatment for KOA, published in Chinese or English journals. Participants were eligible for inclusion if they had a confirmed diagnosis of KOA, with no exclusions based on demographic characteristics such as age, gender, ethnicity, or baseline factors including Kellgren-Lawrence grading. The intervention group was limited to those receiving curcumin exclusively, without restrictions on dosage, formulation, preparation, or administration regimen. Studies were considered eligible if they reported at least one outcome measure, such as the Western Ontario and McMaster Universities Osteoarthritis Index (WOMAC), Visual Analogue Scale (VAS), and adverse events.

Exclusion criteria were applied to omit duplicate publications of the same study, research with incomplete or missing datasets, protocols for systematic reviews or meta-analyses, network meta-analyses, narrative reviews, and studies that had not undergone peer review or formal publication in scholarly journals.

### 2.4 Study selection and data extraction

Two independent researchers conducted the literature search and utilized NoteExpress software to remove duplicate entries. During the initial screening phase, titles and abstracts were examined to exclude studies that were clearly irrelevant. Full texts of the remaining studies were then retrieved and meticulously reviewed in a subsequent round. A data extraction form was developed for SRs and MAs on curcumin for KOA. This form captured essential details such as author names, publication year, number of included studies, sample size, study design, intervention specifics, methodological quality assessment tools, and outcome measures. Any missing information was supplemented by consulting additional sources or by directly contacting the authors via email. The two researchers cross-verified the extracted data, and any discrepancies were resolved through discussions with a third-party adjudicator. A comprehensive list of excluded studies, along with the reasons for their exclusion after full-text review, is provided in the Supplementary Materials.

### 2.5 Assessment of study

Two independent assessors conducted a rigorous appraisal of the methodological rigor, reporting standards, evidence hierarchy, and potential for study duplication among the selected SRs and MAs. Conflicts were resolved by consulting a third-party arbitrator.

The AMSTAR 2 tool was employed to evaluate methodological quality across 16 items, categorizing overall quality as high, moderate, low, or very low (Shea et al., 2017). Reporting quality was scrutinized using the PRISMA 2020 statement (Page et al., 2021). The ROBIS tool was used to assess risk of bias, classifying it as low, high, or uncertain (Whiting et al., 2016). Evidence quality was appraised with the GRADE system, considering several biases and downgrading evidence quality from high to very low accordingly (Guyatt et al., 2008). Study overlap was assessed using the GROOVE tool, which calculates the corrected covered area (CCA) to quantify duplication of primary trials across systematic reviews and meta-analyses. In this approach, N represents the number of included SRs/MAs, r the number of unique primary trials, and c the total number of trial occurrences across all reviews, including duplicates when the same trial was included more than once. The CCA was calculated as (c−r)/[r × (N−1)] and interpreted according to established thresholds: slight (0%–5%), moderate (6%–10%), high (11%–15%), and very high (>15%) overlap (Bracchiglione et al., 2022).

### 2.6 Data synthesize and statistical analysis

We compiled and visually displayed the characteristics and findings of each SR, along with the assessments from AMSTAR 2, PRISMA, ROBIS, GROOVE, and GRADE, in detailed tables and figures for enhanced clarity and comparison.

## 3 Results

### 3.1 Literature selection

**FIGURE 1:**
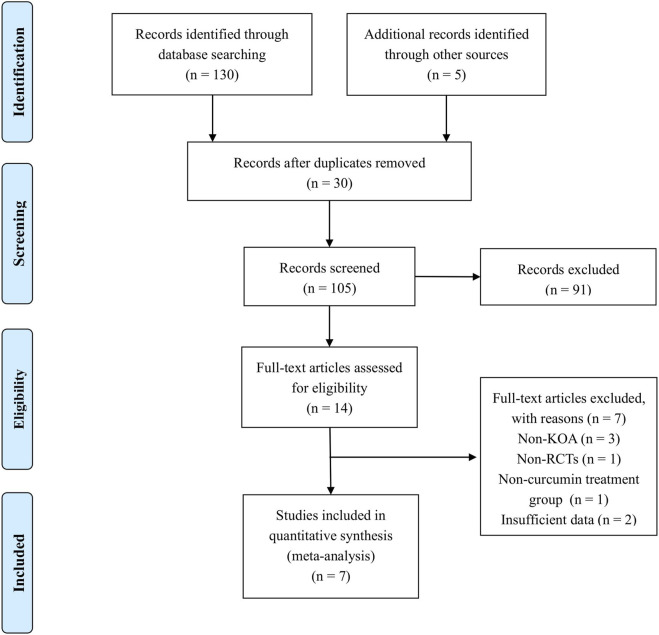
Literature selection procedure.

Based on a predefined search strategy, a total of 135 relevant studies were identified. Following the removal of duplicates, 105 studies were considered for initial review. Upon initial evaluation of article titles and abstracts, 91 studies were deemed ineligible and subsequently removed from consideration. Following a detailed examination of the full texts of the remaining 14 articles, an additional 7 were excluded due to non-compliance with the established inclusion parameters. Ultimately, 7 SRs and MAs that concentrated on the therapeutic application of curcumin in the context of KOA were identified for inclusion in the analysis. The systematic approach to literature curation and the resultant selection outcomes are visually represented in Figure 1.

### 3.2 Basic characteristics of the included SRs/MAs

The data synthesized from the SRs in the document collectively explore the comparative efficacy of curcumin in treating KOA through multiple RCTs carried out from 2017 to 2025. A majority of these trials were conducted in China, with significant contributions also from researchers in Indonesia and the United Kingdom. The sample sizes of these studies varied, ranging from 450 participants in the smallest trial to 1,670 individuals in the largest, providing a comprehensive perspective on the treatment’s effects on diverse patient groups. All studies uniformly employed the Cochrane Risk of Bias Tool to ensure a thorough assessment of the quality and bias in the results, thereby strengthening the reliability of the synthesized outcomes. The primary outcomes evaluated in the studies encompassed the WOMAC scores, which assess pain, stiffness, and physical function, and the VAS, which measures pain intensity. The overall conclusion drawn from the reviews is that curcumin treatments are more effective than placebo and NSAIDs in enhancing the clinical symptoms and quality of life for KOA patients. Specific improvements noted in the studies include substantial pain reduction, enhanced functional mobility, and decreased stiffness. Although some studies reported mild adverse events associated with curcumin, these were typically short-lived and did not diminish the treatment’s benefits. Several reviews also emphasized the need for further research to examine the long-term effects and optimal administration protocols of curcumin to maximize its therapeutic advantages. Six studies have demonstrated that turmeric positively impacts pain and function in KOA, showing greater efficacy than a placebo and similar effectiveness to NSAIDs. All studies found that turmeric did not result in a significantly higher rate of adverse effects compared to the control group. Two studies have summarized serological objective indicators, further supporting turmeric’s positive effects on KOA. The detailed characteristics can be found in Table 1.

**TABLE 1:** Basic characteristics of the included SRs/MAs.

Author (year)	Country	Type of included studies	Number of RCT /Sample size	Number of search databases (English/Chinese)	Intervention		Quality assessment tool	Outcome measures	Overall conclusions
					Treatment Group	Control Group			
Onakpoya et al. (2017)	UK	RCT	7 / 797	(5/0)	Curcuminoids	Placebo/NSAIDs	Cochrane Risk of Bias Tool	VAS score, WOMAC function score, WOMAC pain score, WOMAC stiffness score, WOMAC total score, Use of rescue medication, Lequesne index, Adverse events, Withdrawal rates	Curcuminoids may have some beneficial effects on knee pain and quality of life in patients with knee OA but are less effective at relieving pain compared with ibuprofen. No serious adverse events were reported.
Dai et al. (2021)	China	RCT	10 / 783	(4/0)	Curcuma longa extract	Placebo	Cochrane Risk of Bias Tool	VAS score, WOMAC total score, WOMAC pain score, WOMAC function score, WOMAC stiffness score, Adverse events	Curcuma longa extract is more effective than placebo in pain relief and functional improvement, with no significant difference in adverse events. However, due to heterogeneity in the included studies, more high-quality RCTs with large sample sizes are required to confirm its benefits.
Hsiao et al. (2021)	China	RCT	11 / 1258	(7/1)	Curcuminoids	Placebo/NSAIDs	Cochrane Risk of Bias Tool	VAS score, WOMAC total score, WOMAC pain score, WOMAC function score, WOMAC stiffness score, Adverse events	The results of our meta-analysis suggest that low- and high-dose curcuminoids have similar pain relief effects and AEs in knee OA. Curcuminoids are also associated with better pain relief than NSAIDs; therefore, using curcuminoids as an adjunctive treatment in knee OA is recommended.
Feng et al. (2022)	China	RCT	15 / 1670	(5/4)	Curcuminoids	Placebo/NSAIDs	Cochrane Risk of Bias Tool	VAS score, WOMAC total score, WOMAC pain score, WOMAC function score, WOMAC stiffness score, Adverse events	CURs alone can be expected to achieve considerable analgesic and functional promotion effects for patients with symptomatic knee OA in short term, without inducing an increase of adverse events. However, considering the low quality and substantial heterogeneity of present studies, a cautious and conservative recommendation for broader clinical use of CURs should still be made. Further high-quality studies are necessary to investigate the impact of different dosages, optimization techniques and administration approaches on long-term safety and efficacy of CURs, so as to strengthen clinical decision making for patients with symptomatic knee OA.
Ji et al. (2022)	China	RCT	10 / 783	(4/4)	Curcuma longa extract	Placebo	Cochrane Risk of Bias Tool	VAS score, WOMAC total score, WOMAC pain score, WOMAC function score, WOMAC stiffness score, Adverse events	Current evidence suggests that Curcuma longa extract has more benefit in pain relief and functional improvement for symptomatic knee OA compared to placebo. However, the potential heterogeneity among included studies needs to be considered.
Yu et al. (2025)	China	RCT	10 / 783	(5/2)	Curcuminoids	Placebo/NSAIDs	Cochrane Risk of Bias Tool	VAS score, WOMAC total score, 6 minutes walking distance, CRP, ESR, Adverse events	Curcumin has good anti-inflammatory and analgesic effects, which may be a safer and more effective potential treatment for KOA patients. In the future, large-scale, multi-center and long-term RCTs research can be carried out on more landmark clinical indicators to comprehensively evaluate the feasibility of curcumin in the treatment of KOA.
Hidayat et al. (2025)	Indonesia	RCT	6 / 450	(4/0)	Curcuminoids	Placebo/NSAIDs	Cochrane Risk of Bias Tool	VAS score, WOMAC total score	Curcuma longa benefits knee OA pain and function, being more effective than placebo and comparable to NSAIDs. Despite positive results, limitation and heterogeneity of the studies necessitates further research to explore optimal dosages and administration methods of Curcuma longa as therapeutic option for knee OA.

Abbreviations: WOMAC: Western Ontario and McMaster Universities Osteoarthritis Index, VAS: Visual Analogue Scale,NSAIDs: Non-Steroidal Antiinflammatory Drugs, CRP: C-reactive protein, ESR: Erythrocyte Sedimentation Rate.

### 3.3 Quality evaluation

#### 3.3.1 Methodological evaluation

**FIGURE 2:**
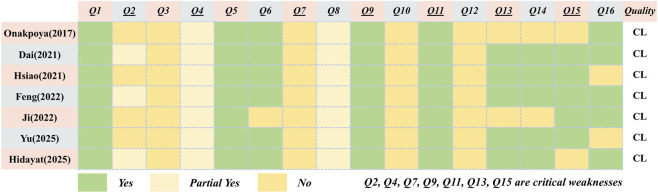
The assessment of AMSTAR 2.

All SRs were rated as having critically poor quality. Regarding critical items, none of the SRs reported the predefined protocols (item 2) or the comprehensive search strategy (item 4). Additionally, no SRs provided a list of excluded literature along with the corresponding reasons (item 7). For item 9, all SRs utilized an appropriate tool to assess the risk of bias for each included study. In terms of item 11, all SRs employed acceptable statistical methods for analyzing the study findings. However, for item 13, two SRs failed to consider the impact of the bias risk of the included literature on evidence integration (Onakpoya et al., 2017; Ji et al., 2022). Regarding item 15, five SRs evaluated publication bias and addressed its potential effects on outcomes (Ji et al., 2022; Hsiao et al., 2021; Yu et al., 2025; Feng et al., 2022; Dai et al., 2021). Five SRs also stated the absence of potential conflicts of interest (Onakpoya et al., 2017; Ji et al., 2022; Feng et al., 2022; Dai et al., 2021; Hidayat et al., 2025). Moreover, items 3 and 10 received the lowest quality assessments. None of the SRs mentioned the reasons for including specific categories of literature or the sources of financing for the included studies. Also, no SRs considered the influence of the bias risk of the included literature on evidence integration. Items 1, 5, and 6 were nearly comprehensively covered. The detailed AMSTAR 2 assessment results are presented in Figure 2.

#### 3.3.2 Reporting quality

**FIGURE 3:**
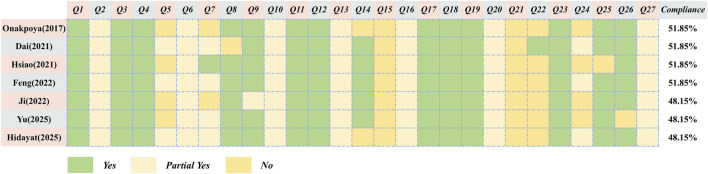
The assessment of PRISMA 2020.

The PRISMA 2020 assessment of the 7 included SRs/MAs showed generally incomplete reporting. Several items (2, 5, 6, 7, 10, 13, 14, 15, 16, 20, 24, and 27) were insufficiently reported, with less than half of the studies providing adequate information for these domains. Detailed item-level results of the PRISMA 2020 checklist are presented in Figure 3.

#### 3.3.3 Risk of bias of included studies

**FIGURE 4:**
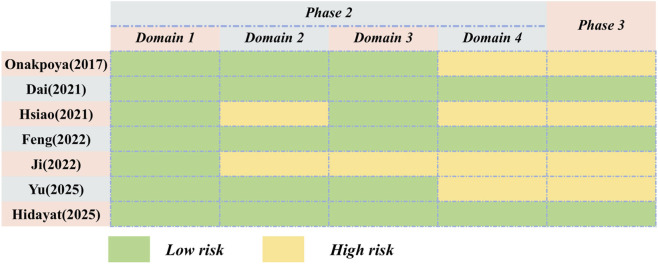
The assessment of ROBIS.

The assessment of the risk of bias in SRs by employing the ROBIS tool led to the following results. In Domain 1 of Phase 2, which focuses on evaluating the study eligibility criteria, all SRs were classified as being at low risk. When it comes to Domain 2, two SRs were judged to be at high risk (Ji et al., 2022; Hsiao et al., 2021), while the remaining ones were at low risk. In Domain 3, one SR was categorized as being at high risk (Ji et al., 2022), and the rest were at low risk. In Domain 4, which is related to the synthesis and findings, three SRs were identified as being at low risk (Feng et al., 2022; Dai et al., 2021; Hidayat et al., 2025). Regarding Phase 3, which is used to evaluate the overall risk of bias in the review, four SRs were determined to be at high risk (Onakpoya et al., 2017; Ji et al., 2022; Hsiao et al., 2021; Yu et al., 2025). The detailed results are illustrated in Figure 4.

#### 3.3.4 Overlap of primary studies

**FIGURE 5:**
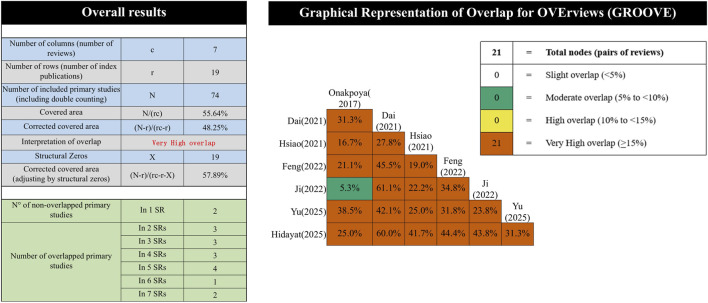
Overlapping of the included SRs.

The GROOVE tool was utilized to assess the overlap among the primary studies included in the literature. The overlap was found among the documents. This tool applies the formula \((N - r)/(rc - r)\) to compute the overlap rate. Among the included studies, there were 21 nodes, and 21 of them exhibited a very high level of overlap. The overall overlap rate was calculated to be 48.25%, suggesting a remarkably high degree of overlap. The overlap structure is further visualized in the overlap matrix provided in Supplementary Figure S1. The detailed assessment results are shown in Figure 5.

#### 3.3.5 Evidence quality grading

The GRADE system was used to evaluate the quality of evidence for 48 outcome measures (as presented in Table 2). Among these measures, VAS and WOMAC score were the most commonly reported ones. The assessment results indicated that a total of 37 outcome measures had critically low quality evidence, 6 had low quality evidence, and only 5 had moderate quality evidence. Notably, there was no outcome measure with high quality evidence. The five factors contributing to the downgrading of evidence quality were as follows: limitations in study design or execution accounted for 100% of the downgrading cases, inconsistency in results was observed in 77.1% of the cases, publication bias was a factor in 45.8% of the cases, imprecision in the data was present in 29.2% of the cases, and indirectness of evidence was found in 0% of the cases.

**TABLE 2:** GRADE evidence quality of included SRs.

Author (year)	Outcomes	N/n	95% CI	I^2^%	Limitations	Inconsistency	Indirectness	Imprecision	Publication bias	Quality of evidence
Onakpoya et al. (2017)	VAS score	5 (366)	MD: -3.30 (-4.99, -2.01)	95	-1①	-2②	0	0	-1④	CL
	WOMAC pain score	3 (167)	MD = -4.42 (-6.64, -2.19)	93	-1①	-2②	0	-1③	-1④	CL
	WOMAC function score	3 (531)	MD: -1.84 (-3.54, -0.13)	98	-1①	-2②	0	0	-1④	CL
	WOMAC stiffness score	3 (531)	MD: 0.10 (-1.08, 1.29)	97	-1①	-2②	0	0	-1④	CL
	WOMAC total score	4 (498)	MD: -3.29 (-6.23, -0.35)	99	-1①	-2②	0	0	-1④	CL
	Quality of life	2 (107)	MD: -2.69 (-3.48, -1.90)	0	-1①	0	0	-1③	-1④	CL
	Use of rescue medication	4 (472)	OR: 0.52 (0.29, 0.90)	52	-1①	-2②	0	0	-1④	CL
	Adverse events	5 (727)	OR: 0.84 (0.66, 1.08)	0	-1①	0	0	0	-1④	L
	Withdrawal	6 (797)	OR: 0.93 (0.62, 1.41)	13	-1①	-1②	0	0	-1④	CL
Dai et al. (2021)	VAS score	8 (569)	MD: −2.21 (−3.15, −1.28)	94	-1①	-2②	0	0	0	CL
	WOMAC total score	5 (377)	MD: -11.93 (-16.63, -7.23)	81	-1①	-2②	0	0	0	CL
	WOMAC pain score	6 (547)	MD: -1.94 (-2.80, -1.09)	76	-1①	-2②	0	0	0	CL
	WOMAC function score	6 (547)	MD: -6.45 (-9.10, -3.80)	83	-1①	-2②	0	0	0	CL
	WOMAC stiffness score	6 (547)	MD: -0.53 (-0.95, -0.11)	77	-1①	-2②	0	0	0	CL
	Adverse events	7 (623)	OR: 1.08 (0.69, 1.70)	19	-1①	0	0	-1③	0	L
Hsiao et al. (2021)	VAS score	7 (757)	MD: -1.66 (-3.37, 0.06)	96	-1①	-2②	0	-1③	-1④	CL
	WOMAC total score	6 (731)	MD: -0.80 (-1.73, 0.14)	90.8	-1①	-2②	0	-1③	0	CL
	WOMAC pain score	6 (834)	MD: -0.79 (-1.41, -0.16)	90	-1①	-2②	0	-1③	0	CL
	WOMAC function score	6 (834)	MD: -1.50 (-3.59, 0.58)	98	-1①	-2②	0	-1③	0	CL
	WOMAC stiffness score	6 (834)	MD: -1.20 (-3.23, 0.83)	97	-1①	-2②	0	-1③	0	CL
	Adverse events	9 (1150)	OR: 0.77 (0.44, 1.34)	42	-1①	-1②	0	-1③	0	CL
Feng et al. (2022)	VAS score	11 (870)	MD: -1.77 (-2.44, -1.09)	87	-1①	-2②	0	0	0	CL
	WOMAC total score	7 (795)	MD: -10.47 (-15.65, -5.29)	0	-1①	0	0	0	0	M
	WOMAC pain score	8 (956)	MD: -1.94 (-2.91, -0.97)	79	-1①	-2②	0	0	0	CL
	WOMAC function score	8 (956)	MD: -6.36 (-8.94, -3.78)	79	-1①	-2②	0	0	0	CL
	WOMAC stiffness score	8 (956)	MD: -0.54 (-1.03, -0.05)	78	-1①	-2②	0	0	0	CL
	Adverse events	9 (969)	OR: 1.07 (0.70, 1.65)	33	-1①	-1②	0	0	0	L
Ji et al. (2022)	VAS score	8 (616)	MD: -0.59 (-1.03, -0.15)	99	-1①	-2②	0	0	0	CL
	WOMAC total score	4 (282)	MD: -11.73 (-16.93, -6.54)	88	-1①	-2②	0	0	-1④	CL
	WOMAC pain score	4 (382)	MD: -1.52 (-2.68, -0.36)	96	-1①	-2②	0	0	-1④	CL
	WOMAC function score	4 (382)	MD: -5.28 (-7.73, -2.84)	99	-1①	-2②	0	0	-1④	CL
	WOMAC stiffness score	4 (382)	MD: -0.89 (-1.29, -0.49)	86	-1①	-2②	0	0	-1④	CL
	Adverse events	9 (662)	OR: 1.19(0.74, 1.90)	0	-1①	0	0	-1③	-1④	CL
Yu et al. (2025)	VAS score	5 (419)	MD: 5.44 (2.74, 8.14)	100	-1①	-2②	0	0	-1④	CL
	WOMAC total score	7 (913)	MD: 5.04 (1.91, 8.17)	99	-1①	-2②	0	0	-1④	CL
	6 minutes walking distance	2 (417)	MD: -8.29 (-13.38, -3.20)	24	-1①	0	0	0	-1④	L
	CRP	3 (291)	MD: 1.48 (-0.03, 3.00)	97	-1①	-2②	0	-1③	-1④	CL
	ESR	3(191)	MD: 0.79, (-0.51,2.08)	89	-1①	-2②	0	-1③	-1④	CL
	Adverse events	10 (1233)	OR: 1.5 (1.10, 2.05)	49	-1①	-1②	0	0	0	L
Hidayat et al. (2025)	VAS score	6 (448)	MD: 18.25 (-7.79, 28.72)	99	-1①	-2②	0	-1③	0	CL
	WOMAC total score	2 (107)	MD: 8.12 (2.11, 18.35)	98	-1①	-2②	0	0	-1④	CL
Curcuminoids vs NSAID										
Feng et al. (2022)	VAS score	3 (272)	MD: -0.30 (-0.63, 0.04)	6	-1①	0	0	0	0	M
	WOMAC total score	3(517)	MD: -0.68 (-3.88, 2.52)	0	-1①	0	0	0	0	M
	WOMAC pain score	2 (475)	MD: -0.24 (-0.47, 0.96)	0	-1①	0	0	0	0	M
	WOMAC function score	2 (475)	MD: -0.57 (-3.07, 1.94)	0	-1①	0	0	0	0	M
	WOMAC stiffness score	2 (475)	MD: 0.19 (-0.17, 0.56)	0	-1①	0	0	-1③	0	L
	Adverse events	5 (800)	MD: 0.65 (0.41, 1.03)	56	-1①	-2②	0	0	0	CL
Hidayat et al. (2025)	WOMAC total score	2 (159)	MD: 11.99 (3.92, 15.23)	80	-1①	-2②	0	0	-1④	CL

Abbreviations: CL: critically low, L: low, M: moderate, NSAIDs: Non-Steroidal Antiinflammatory Drugs, ①: The design of the experiment with a large bias in random, distributive hiding or blind;②: The confidence interval overlaps less, the heterogeneity test *P* is Critically small, and the *I* ^*2*^ is larger;③: Confidence interval is not narrow enough;④: Asymmetric funnel plot or fewer studies are included and there may be greater publication bias.

## 4 Discussion

### 4.1 Major study findings

To the best of our knowledge, this is the first comprehensive review of SRs assessing curcumin’s effectiveness and safety for KOA. We applied AMSTAR 2, ROBIS, PRISMA, GROOVE, and GRADE tools to evaluate these SRs. AMSTAR 2 identified multiple major flaws in all SRs, resulting in very low-quality ratings. PRISMA-based reporting quality was poor, and ROBIS ratings indicated a potential misrepresentation of evidence in four SRs. However, GRADE evaluations suggested that curcumin improves joint symptoms and physical quality of life in KOA patients, with a favorable safety profile. Despite the suggested benefits, the combined review evidence was insufficient for definitive conclusions, advising caution in recommending curcumin for KOA.

### 4.2 Implications for further study

The findings from the AMSTAR 2 assessment, the PRISMA checklist, and the ROBIS tool suggest that there is a considerable need for improvement in both the methodological and reporting standards of SRs. Researchers should ensure pre-registration with PROSPERO, carefully plan their studies, and promptly address potential biases to minimize their impact. It is essential to conduct an exhaustive literature review that includes grey literature, manual searches, and considers language barriers, while also taking into account the propensity for the publication of positive results. A clear list of excluded literature should be provided to enhance the credibility and allow for better evaluation of the evidence quality. Researchers must clearly justify their choices of study types included to ensure that inclusion criteria are appropriate. Disclosure of potential funding sources and conflicts of interest in the original studies, as well as the role of funders in the research process, is crucial for maintaining transparency. The risk of bias in the included literature should be thoroughly evaluated. For studies showing significant heterogeneity, researchers should perform subgroup and sensitivity analyses to identify and explain the sources of this heterogeneity, thereby increasing the reliability of the combined results. The review identified a large variance in intervention protocols among the included SRs, attributed to differences in the types of curcumin, treatment durations, and doses, which significantly affect outcomes. Future clinical researchers must comply with reporting standards to accurately detail the intervention protocols. Users of the evidence should consider how these factors might influence the outcomes and reporting of studies and recognize that the actual trial designs and operations might meet the evaluation criteria but fail to be reported, leading to lower evaluation scores. The ROBIS tool particularly highlights flaws or limitations in the design, execution, and analysis of SRs more than AMSTAR 2 and PRISMA. Using AMSTAR 2 and PRISMA in conjunction with ROBIS provides a more comprehensive evaluation of SRs, with each tool offering complementary perspectives and insights (Gómez-García et al., 2017).

Utilizing the GROOVE software, a comprehensive analysis of 21 studies revealed an extensive overlap of 48.25%, suggesting a notable prevalence of redundant data within the existing body of research. Such substantial overlap has the potential to introduce bias into systematic reviews and meta-analyses by amplifying the influence of frequently re-analyzed data points across various studies. Addressing this redundancy is essential for maintaining the integrity of meta-analytic outcomes. Strategies to mitigate the impact of high overlap include conducting sensitivity analyses to gauge the influence of individual studies on aggregate findings or selectively excluding studies with substantial overlap from certain analyses to reduce the risk of bias. While overlap does not automatically diminish the credibility of the evidence, it raises questions about the breadth and representativeness of the studies included in the synthesis. A more diverse and expansive range of studies is generally more likely to produce robust and widely applicable conclusions compared to a set characterized by significant overlap. The implications of these findings highlight the necessity for future research to broaden its scope in terms of participant demographics, data sources, and methodological approaches. By diversifying the research landscape, the field can expand the evidence base, minimize redundancy, and ultimately enhance the reliability and applicability of its conclusions.

The SRs on the efficacy and safety of curcumins for KOA report generally positive effects. However, the evidence quality was notably low, primarily due to inadequate reliability assessments of individual outcomes. The GRADE system indicated that the evidence quality for five outcomes was moderate, while most were rated as low or very low, with none ranked high. Consequently, the reliability of SRs as clinical guidelines is questionable due to potential biases that might distort the true effects. Key drawbacks stem from deficiencies in the design and execution of the original studies, such as poor randomization, inadequate blinding, and insufficient concealment of allocation. Publication bias was prevalent, noted in 77.1% of cases, followed by issues of imprecision (45.8%) and inconsistency (29.2%). The limited number of patients and events in these studies led to broad confidence intervals, which further compromised the evidence quality for comparisons like PRP versus HA in treating KOA. Moreover, some SRs did not conduct thorough literature searches, missing unpublished studies with negative results, which inflated the publication bias concerning outcomes. Variations in the curcumin treatments among studies—differing in components, doses, intervals, and application frequencies—added to the heterogeneity. This inconsistency necessitated downgrading the evidence and highlighted the need for careful subgroup analyses to interpret variations effectively. The overall quality of evidence from SRs depends heavily on the primary studies’ methodological rigor. To enhance future clinical research, methodological training for researchers should be emphasized. Further, conducting quality analyses and evaluations of primary studies in this field could improve the quality of clinical trials and standardize SR methodologies, creating a stronger foundation for evidence-based medicine.

### 4.3 Comparison with previous umbrella review

A recent umbrella meta-analysis by Bideshki et al. quantitatively synthesized 11 meta-analyses published up to September 2023 (Bideshki et al., 2024). Their pooled analyses concluded that curcumin supplementation significantly reduced pain and improved function in knee osteoarthritis, supporting its clinical efficacy. Our review differs from theirs in several important respects. First, we searched eight databases up to January 2025, thereby including more recent systematic reviews and meta-analyses. Second, instead of repeating a quantitative synthesis, we systematically re-evaluated each SR/MA in terms of methodological quality (AMSTAR-2, ROBIS), reporting completeness (PRISMA 2020), and evidence certainty (GRADE), presenting item-level results in supplementary materials. Third, we explicitly quantified study overlap using the GROOVE tool (CCA = 48.25%) and provided an overlap matrix, revealing substantial duplication of primary trials across reviews. Based on these findings, we deliberately reframed our study as a rather than a second quantitative umbrella meta-analysis. Consequently, our conclusions are more cautious: while curcumin shows potential clinical benefit, the certainty of the evidence remains low to very low, underscoring the urgent need for rigorously designed and transparently reported RCTs and SRs in this field.

### 4.4 Limitations

This study has several limitations. First, our literature search was limited to publications in English and Chinese, and although we manually included grey literature, relevant studies in other languages may have been omitted, potentially introducing language or publication bias. Second, we reframed our work as a critical overview rather than conducting a new quantitative umbrella meta-analysis. This decision was made to avoid duplicate counting of primary trials, which we identified as substantial (corrected covered area = 48.25%) using the GROOVE tool. While this approach enhances methodological transparency, it also means that we did not provide updated pooled effect estimates beyond those already published in previous umbrella meta-analyses. Third, although we systematically evaluated methodological quality (AMSTAR-2), reporting completeness (PRISMA 2020), and risk of bias (ROBIS), as well as evidence certainty (GRADE), these assessments are inherently subjective to some degree, particularly in judgments regarding downgrading decisions. Fourth, the included SRs/MAs varied in their handling of curcumin formulations, dosages, and treatment durations, and most did not conduct appropriate subgroup analyses. This heterogeneity, coupled with generally low methodological rigor, reduces the certainty of the evidence base. Finally, although we uploaded comprehensive supplementary files (including search strategies, excluded studies list, and item-level appraisal tables), our PROSPERO registration was limited and did not prespecify all analytical details, which should be addressed in future protocol planning.

## 5 Conclusion

Curcumins may be a safe, effective treatment for KOA, but existing studies lack robust quality, complicating guideline development. Clinicians should cautiously use this evidence. Future efforts should focus on multicentric, randomized trials to improve design and bias control and standardize efficacy indices. SR authors need to follow strict quality criteria to enhance SR quality and support high-quality evidence-based medicine.

## Data Availability

The original contributions presented in the study are included in the article/Supplementary Material, further inquiries can be directed to the corresponding authors.
