# Supplementary figures and images for: A critical review of systematic reviews and meta-analyses of curcumin for knee osteoarthritis
Source: Front Pharmacol. 2026 Jan 5;16:1664319. doi: 10.3389/fphar.2025.1664319 (PMC12813030; doi:10.3389/fphar.2025.1664319)

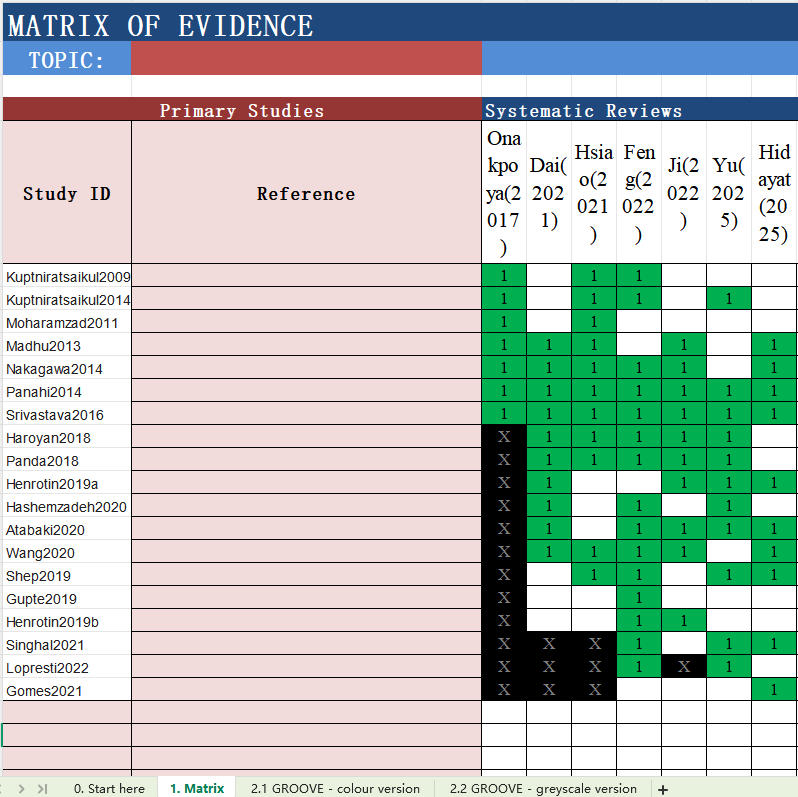

Supplement: Supplementary file 1 [file Image1.jpeg]
